# Supplementary material for: Genomes of a Novel Group of Phages That Use Alternative Genetic Code Found in Human Gut Viromes
Source: Int J Mol Sci. 2023 Oct 18;24(20):15302. doi: 10.3390/ijms242015302 (PMC10607447; doi:10.3390/ijms242015302)
Supplement: Supplementary file 1 [file ijms-24-15302-s001.zip › Data S1 Babkin et al.pdf]

Data S1: Annotation of the phAss genome translated using TAG stop codon recoding

# ORF	Coordinates	Putative product	Suppressor TAG stop codone
1	287-33	hypothetical protein	
2	465-343	hypothetical protein	
3	804-577	hypothetical protein	
4	1710-1162	hypothetical protein	
5	2543-1782	N-acetylmuramoyl-L-alanine amidase	
6	2856-2566	hypothetical protein	
7	3013-2852	hypothetical protein	
8	3132-3016	hypothetical protein	
9	3291-3139	hypothetical protein	
10	3476-3297	hypothetical protein	
11	3597-4640	hypothetical protein	+
12	4667-5224	hypothetical protein	+
13	5261-14710	phage tail tape measure protein	+
14	14730-21197	hypothetical protein	+
15	21853-21221	hypothetical protein	+
16	22039-23202	terminase small subunit	+
17	23189-25006	terminase large subunit	+
18	25070-25456	endonuclease	+
19	25474-27108	portal protein	+
20	27032-27505	hypothetical protein	+
21	27524-33163	Ig domain containing protein	+
22	33178-35400	putative flagellin-like protein	+
23	35615-37441	hypothetical protein	
24	37499-38062	hypothetical protein	
25	38085-39119	major capsid protein	
26	39160-39645	hypothetical protein	
27	39650-40099	hypothetical protein	+
28	40120-40404	hypothetical protein	
29	40419-41393	hypothetical protein	+
30	41414-41983	hypothetical protein	
31	42010-42747	tail-to-head joining protein	+
32	42820-44433	tail fiber protein	
33	44661-45947	receptor-binding tail fiber protein	
34	46674-46021	hypothetical protein	
35	47148-46957	hypothetical protein	
36	51134-47286	DNA polymerase III, alpha subunit	
37	52585-51152	ATP-dependent DNA helicase	
38	53925-52588	DNA primase	
39	54791-53952	single-strand binding protein	
40	55807-54809	AAA domain RecA protein	
41	57447-55879	single-stranded-DNA-specific exonuclease RecJ p	+
42	58190-57684	crossover junction endodeoxyribonuclease RuvC	
43	58840-58190	thymidylate synthase complementing protein	+
44	59061-58843	hypothetical protein	
45	59665-59069	ATP-dependent Clp protease	+
46	59923-59687	ribonucleoside-triphosphate reductase activatin	
47	62379-60193	ribonucleotide reductase of class III (anaerobic), large subunit	
48	62532-62442	1tRNA-SUP-CTA	
49	62610-62536	2tRNA-SUP-CTA	
50	62703-62617	3tRNA-Leu-TAA	
51	63005-62721	multiple antibiotic resistance protein MarR/DNA	+
52	63985-63008	peptidoglycan endopeptidase/GIY-YIG nuclease	+

53	65936-64062	DNA gyrase	
54	67749-65953	DNA topoisomerase IV B-subunit	
55	68352-67831	hypothetical protein	+
56	69546-68356	sulfatase-maturating enzyme	+
57	70558-69536	sulfatase-maturating enzyme/aldolase class I	+
58	71093-70515	hypothetical protein	+
59	71385-71164	hypothetical protein	
60	72278-71394	N-acetylmuramoyl-L-alanine amidase	+
61	73106-72390	lysozyme	
62	73981-73142	PhoH family ribonuclease	
63	74225-74067	hypothetical protein	
64	74636-74238	phage holin	+
65	75098-74658	putative membrane-bound protein	+
66	75540-75094	putative spike protein	+
67	76377-75634	pilus assembly protein	+
68	81417-76420	capsid protein	+
69	82455-81451	L-shaped tail fiber protein	+
70	83110-82472	L-shaped tail fiber protein	
71	84318-83329	hypothetical protein	
72	85208-84675	hypothetical protein	
73	86058-85204	bifunctional 5,10-methylene-tetrahydrofolate	
74	86796-86098	dUTPase	+
75	87583-86813	exodeoxyribonuclease III	
76	88751-87687	ATP-dependent DNA ligase	
77	88971-88780	putative ATP synthase	
78	89729-89052	formate/nitrite transporter family protein	
79	90193-89729	hypothetical protein	+
80	91015-90557	NADAR family protein	+
81	91469-90999	antitermination protein, Q-dependent.	+
82	92043-91351	hypothetical protein	+
83	93043-92747	hypothetical protein	
84	93703-93221	TFIIB zinc-binding	
85	94213-93710	hypothetical protein	
86	96250-94307	hypothetical protein	
87	96610-96269	hypothetical protein	
88	96724-96634	4tRNA-Ser-GCT	
89	97783-96863	glutamine dependent NAD+ synthetase	
90	98202-97786	polynucleotide kinase	
91	99794-98199	nicotinamide phosphoribosyltransferase	
92	100756-99794	ribose-phosphate pyrophosphokinase	
93	101298-100756	nicotinamidase	
94	101818-101540	amine/thiol ligase	
95	102221-101838	hypothetical protein	
96	102331-102254	5tRNA-Glu-TTC	
97	102635-102559	6tRNA-Asp-GTC	
98	102840-102758	7tRNA-Leu-GAG	
99	103091-103003	8tRNA-Leu-CAA	
100	103237-103160	9tRNA-Leu-CAG	
101	103457-103384	10tRNA-Gln-CTG	
102	103627-103463	hypothetical protein	
103	103922-103686	hypothetical protein	
104	104173-103922	hypothetical protein	
105	104269-104193	11tRNA-Arg-ACG	
106	104500-104412	12tRNA-Ser-GGA	
107	104727-104653	13tRNA-His-GTG	
108	105005-104929	14tRNA-Lys-CTT	

109	105219-105144	15tRNA-Phe-GAA	
110	105316-105240	16tRNA-Arg-TCT	
111	105562-105329	phosphocarrier protein	
112	105669-105593	17tRNA-Cys-GCA	
113	105820-105744	18tRNA-Sup-CTA	
114	105989-105912	19tRNA-Glu-CTC	
115	106405-106329	20tRNA-Thr-TGT	
116	107044-106968	21tRNA-Val-TAC	
117	107377-107301	22tRNA-Leu-TAG	
118	107470-107379	23tRNA-Ser-TGA	
119	107722-107477	hypothetical protein	
120	108030-107737	hypothetical protein	
121	108198-108046	hypothetical protein	
122	108296-108221	24tRNA-Ala-TGC	
123	108429-108352	25tRNA-Asn-GTT	
124	108602-108527	26tRNA-Pro-GGG	
125	108681-108606	27tRNA-Pro-TGG	
126	108996-108742	transcription factor	
127	109212-109126	hypothetical protein	
128	109318-109242	28tRNA-Met-CAT	
129	109518-109442	29tRNA-Met-CAT	
130	110132-109635	hypothetical protein	
131	110224-110150	30tRNA-Trp-CCA	
132	110558-110482	31tRNA-Ile-GAT	
133	110862-110787	32tRNA-Gly-GCC	
134	111316-110930	hypothetical protein	
135	111866-111312	lipoprotein	
136	111972-111896	33tRNA-Gly-TCC	
137	112328-112253	34tRNA-Lys-TTT	
138	112722-112450	hypothetical protein	
139	112942-112718	hypothetical protein	
140	113383-113021	hypothetical protein	
141	113879-113403	hypothetical protein	+
142	114007-113931	35tRNA-Met-CAT	
143	114300-114225	36tRNA-Gln-TTG	
144	114817-114464	hypothetical protein	
145	115738-114911	nucleotidyltransferase-like protein	
146	116289-115951	hypothetical protein	
147	116812-116294	CYTH-like phosphatases	
148	117145-116825	hypothetical protein	
149	117579-117148	hypothetical protein	
150	118322-117744	metallophosphoesterase family protein	
151	118807-118322	hypothetical protein	+
152	119164-118862	hypothetical protein	
153	119492-119241	hypothetical protein	
154	120041-119508	P-loop containing nucleoside triphosphate hydrolase	+
155	120321-120037	hypothetical protein	
156	120599-120321	hypothetical protein	
157	120873-120604	hypothetical protein	
158	122264-120969	putative peptidoglycan endopeptidase	
159	123462-122485	hypothetical protein	+
160	123841-123479	hypothetical protein	
161	124552-124307	hypothetical protein	
162	125293-124844	hypothetical protein	
163	126656-125814	hypothetical protein	+
164	126903-126691	3'-5' exonuclease	

165	127659-126958	exoribonuclease
166	128143-127727	hypothetical protein
167	128591-128181	hypothetical protein
168	128841-128644	hypothetical protein
169	129105-128875	hypothetical protein
170	129735-129373	hypothetical protein
171	129947-129738	hypothetical protein
172	130497-130036	phosphatase
173	130787-130530	hypothetical protein
174	131639-130863	RNAse
175	132151-131741	hypothetical protein
176	132874-132203	hypothetical protein
177	133499-133071	hypothetical protein
178	134124-133585	hypothetical protein
179	134568-134212	hypothetical protein
180	134803-134648	hypothetical protein
181	134964-134809	hypothetical protein
182	135369-134998	hypothetical protein
183	135874-135578	hypothetical protein
