# Supplementary material for: Genomes of a Novel Group of Phages That Use Alternative Genetic Code Found in Human Gut Viromes
Source: Int J Mol Sci. 2023 Oct 18;24(20):15302. doi: 10.3390/ijms242015302 (PMC10607447; doi:10.3390/ijms242015302)
Supplement: Supplementary file 1 [file ijms-24-15302-s001.zip › Data S2 Babkin et al.pdf]

Data S2: Annotation of the ctckW2 genome translated using TAG stop codon recoding

# ORF	Coordinates	Putative product	Suppressor TAG stop codone
1	278-30	hypothetical protein	
2	490-362	hypothetical protein	
3	828-574	hypothetical protein	
4	1370-1038	hypothetical protein	+
5	2222-1596	N-acetylmuramoyl-L-alanine amidase	
6	2588-2484	hypothetical protein	
7	2809-2588	hypothetical protein	
8	3249-4283	hypothetical protein	+
9	4303-4863	hypothetical protein	+
10	4896-14348	phage tail tape measure protein	+
11	14365-14856	hypothetical protein	+
12	14972-20935	hypothetical protein	+
13	21588-20953	hypothetical protein	+
14	21774-22940	terminase small subunit	+
15	22840-24744	terminase large subunit	+
16	24805-25194	endonuclease	+
17	25209-26846	portal protein	+
18	26865-27242	hypothetical protein	+
19	27258-32900	Ig domain containing protein	+
20	32912-35137	putative flagellin-like protein	+
21	35349-37175	hypothetical protein	
22	37230-37796	hypothetical protein	
23	37816-38853	major capsid protein	
24	38891-39379	hypothetical protein	
25	39381-39833	hypothetical protein	+
26	39851-40138	hypothetical protein	+
27	40150-41127	hypothetical protein	+
28	41145-41717	hypothetical protein	
29	41741-42481	tail-to-head joining protein	+
30	42551-45760	receptor-binding tail fiber protein	
31	46489-45833	hypothetical protein	
32	47108-46785	hypothetical protein	
33	50949-47098	DNA polymerase III, alpha subunit	
34	52400-50964	ATP-dependent DNA helicase	
35	53740-52400	DNA primase	
36	54606-53764	Single-strand binding protein	
37	55622-54621	AAA domain RecA	
38	57268-55691	single-stranded-DNA-specific exonuclease RecJ p	+
39	58005-57496	crossover junction endodeoxyribonuclease RuvC	
40	58655-58002	thymidylate synthase complementing protein	+
41	58876-58655	hypothetical protein	
42	59420-58881	ATP-dependent Clp protease proteolytic subunit	
43	60008-59499	ribonucleoside-triphosphate reductase activatin	+
44	62194-60005	ribonucleotide reductase of class III	
45	62347-62260	tRNA-Sup-CTA	
46	62425-62354	tRNA-Sup-CTA	
47	62518-62435	tRNA-Leu-TAA	
48	62814-62533	multiple antibiotic resistance protein MarR/DNA	
49	63800-62820	peptidoglycan endopeptidase/GIY-YIG nuclease fa	+
50	65751-63874	DNA gyrase/topoisomerase IV, subunit A	
51	67564-65765	DNA topoisomerase IV B-subunit	
52	68518-67643	hypothetical protein	+

53	69600-68455	sulfatase-maturating enzyme	+
54	70829-69606	sulfatase-maturating enzyme	+
55	71117-70905	hypothetical protein	
56	72004-71123	N-acetylmuramoyl-L-alanine amidase	+
57	72832-72113	Soluble lytic murein transglycosylase, lysozyme	
58	73707-72865	PhoH family protein, ribonuclease and ATPase	+
59	73951-73790	hypothetical protein	
60	74329-73961	phage holin	
61	74809-74366	putative membrane-bound protein	+
62	75251-74802	putative spike protein	+
63	76091-75342	pilus assembly protein	+
64	81311-76131	capsid protein	+
65	82349-81342	L-shaped tail fiber protein	
66	83004-82363	L-shaped tail fiber protein	
67	84212-83220	hypothetical protein	
68	85033-84536	hypothetical protein	
69	85277-85080	hypothetical protein	
70	85668-85348	hypothetical protein	
71	85895-85629	bifunctional 5,10-methylene-tetrahydrofolate	
72	86621-85932	dUTPase	
73	87408-86635	Exodeoxyribonuclease III	
74	88603-87497	ATP-dependent DNA ligase	
75	88790-88590	putative ATP synthase	
76	89543-88863	formate/nitrite transporter family protein	
77	89854-89540	hypothetical protein	
78	90185-89952	hypothetical protein	
79	90318-90175	hypothetical protein	
80	90830-90387	NADAR family protein	
81	91170-90811	antitermination protein, Q-dependent	
82	91843-91163	hypothetical protein	+
83	92877-92587	hypothetical protein	
84	93498-93013	TFIIB zinc-binding	
85	94008-93502	hypothetical protein	
86	96042-94099	hypothetical protein	
87	96402-96058	hypothetical protein	
88	96516-96429	tRNA-Ser-GCT	
89	97574-96648	glutamine dependent NAD+ synthetase	
90	98069-97575	polynucleotide kinase	
91	99573-97933	nicotinamide phosphoribosyltransferase	
92	100535-99570	ribose-phosphate pyrophosphokinase	
93	101077-100532	nicotinamidase	
94	101596-101315	probable ATP-dependent amine/thiol ligase family	
95	101999-101613	hypothetical protein	
96	102109-102035	tRNA-Glu-TTC	
97	102420-102347	tRNA-Asp-GTC	
98	102625-102546	tRNA-Leu-GAG	
99	103024-102950	tRNA-Leu-CAG	
100	103244-103174	tRNA-Gln-CTG	
101	103414-103247	hypothetical protein	
102	103695-103456	hypothetical protein	
103	103946-103692	hypothetical protein	
104	104042-103969	tRNA-Arg-ACG	
105	104500-104429	tRNA-His-GTG	
106	104978-104906	tRNA-Phe-GAA	
107	105076-105003	tRNA-Arg-TCT	
108	105322-105086	phosphocarrier protein HPr	

109	105621-105547	tRNA-Glu-CTC	
110	106066-105986	tRNA-Thr-TGT	
111	106673-106600	tRNA-Ile-GAT	
112	106855-106679	hypothetical protein	
113	106955-106882	tRNA-Leu-TAG	
114	107346-107050	hypothetical protein	
115	107447-107375	tRNA-Ala-TGC	
116	107580-107506	tRNA-Asn-GTT	
117	107753-107674	tRNA-Pro-GGG	
118	107832-107760	tRNA-Pro-TGG	
119	108147-107890	hypothetical protein	
120	108441-108274	hypothetical protein	
121	108596-108523	tRNA-Met-CAT	
122	109210-108710	hypothetical protein	
123	109302-109231	tRNA-Trp-CCA	
124	109636-109563	tRNA-Ile-GAT	
125	110390-110001	hypothetical protein	
126	110937-110383	hypothetical protein	
127	111042-110969	tRNA-Gly-TCC	
128	111396-111324	tRNA-Lys-TTT	
129	111786-111511	hypothetical protein	
130	112006-111779	hypothetical protein	
131	112432-112067	hypothetical protein	
132	112792-112436	hypothetical protein	
133	113051-112978	tRNA-Met-CAT	
134	113130-113058	tRNA-Gln-TTG	
135	113429-113163	hypothetical protein	
136	113698-113474	hypothetical protein	+
137	114080-113622	hypothetical protein	+
138	114974-114150	hypothetical protein	
139	115531-115190	hypothetical protein	
140	116054-115533	CYTH-like phosphatases	
141	116375-116064	hypothetical protein	
142	116809-116375	hypothetical protein	
143	116996-116811	hypothetical protein	
144	117558-116971	metallophosphoesterase family protein	
145	117860-117555	hypothetical protein	
146	118252-117920	hypothetical protein	
147	118612-118358	hypothetical protein	
148	119161-118625	hypothetical protein	+
149	119651-119370	hypothetical protein	
150	119928-119653	hypothetical protein	
151	121241-120021	putative peptidoglycan endopeptidase	
152	122439-121459	hypothetical protein	+
153	123534-123238	hypothetical protein	
154	124200-123748	hypothetical protein	
155	124632-124474	hypothetical protein	
156	125513-124701	hypothetical protein	
157	126179-125742	hypothetical protein	
158	126917-126231	hypothetical protein	
159	127419-127000	hypothetical protein	
160	127866-127453	hypothetical protein	
161	128115-127876	hypothetical protein	
162	128379-128146	hypothetical protein	
163	128603-128415	hypothetical protein	+
164	129352-128888	hypothetical protein	

165	130479-129739	hypothetical protein
166	130967-130554	hypothetical protein
167	131438-131016	hypothetical protein
168	131813-131568	hypothetical protein
169	132381-132007	hypothetical protein
170	132787-132587	hypothetical protein
