# Supplementary material for: Genomes of a Novel Group of Phages That Use Alternative Genetic Code Found in Human Gut Viromes
Source: Int J Mol Sci. 2023 Oct 18;24(20):15302. doi: 10.3390/ijms242015302 (PMC10607447; doi:10.3390/ijms242015302)
Supplement: Supplementary file 1 [file ijms-24-15302-s001.zip › Data S3 Babkin et al.pdf]

Data S3: Annotation of the ct6IQ4 genome translated using TAG stop codon recoding

# ORF	Coordinates	Putative product	Suppressor TAG stop codone
1	287-30	hypothetical protein	
2	706-362	hypothetical protein	
3	1338-1129	hypothetical protein	
4	2189-1347	endo-beta-N-acetylglucosaminidase	
5	2352-2182	hypothetical protein	
6	2624-2469	hypothetical protein	
7	2887-2624	hypothetical protein	
8	3063-2881	hypothetical protein	
9	3184-4386	hypothetical protein	+
10	4410-5003	hypothetical protein	+
11	5023-14652	phage tail tape measure protein	+
12	14669-21160	hypothetical protein	+
13	21813-21178	hypothetical protein	+
14	21999-23165	terminase small subunit	+
15	23065-24969	terminase large subunit	+
16	25030-25419	endonuclease	+
17	25434-27071	portal protein	+
18	27487-33234	Ig domain containing protein	+
19	33248-35407	putative flagellin-like protein	+
20	35618-37441	hypothetical protein	
21	37496-38062	hypothetical protein	
22	38082-39119	major capsid protein	
23	39157-39645	hypothetical protein	
24	39647-40099	hypothetical protein	
25	40117-40404	hypothetical protein	
26	40416-41393	hypothetical protein	+
27	41411-41983	hypothetical protein	
28	42007-42747	tail-to-head joining protein	+
29	42817-46026	receptor-binding tail fiber protein	
30	46754-46098	hypothetical protein	
31	47237-47010	hypothetical protein	
32	51214-47363	DNA polymerase III, alpha subunit	
33	52665-51229	ATP-dependent DNA helicase	
34	54005-52665	DNA primase	
35	54871-54029	single-strand binding protein	
36	55887-54886	AAA domain RecA	
37	57533-55956	single-stranded-DNA-specific exonuclease RecJ p	+
38	58270-57761	crossover junction endodeoxyribonuclease RuvC	
39	58920-58267	thymidylate synthase complementing protein	+
40	59141-58920	hypothetical protein	
41	59745-59146	ATP-dependent Clp protease proteolytic subunit	+
42	60003-59764	ribonucleoside-triphosphate reductase activatin	
43	62459-60270	ribonucleotide reductase of class III	
44	62612-62525	tRNA-Sup-CTA	
45	62690-62619	tRNA-Sup-CTA	
46	62783-62700	tRNA-Leu-TAA	
47	63085-62798	multiple antibiotic resistance protein MarR/DNA	
48	64065-63085	peptidoglycan endopeptidase/GIY-YIG nuclease fa	+
49	66016-64139	DNA gyrase/topoisomerase IV, subunit A	

50	67829-66030	DNA topoisomerase IV B-subunit	
51	68432-67908	hypothetical protein	+
52	69626-68433	sulfatase-maturating enzyme	+
53	70638-69613	sulfatase-maturating enzyme/aldolase class I	+
54	71173-70592	hypothetical protein	+
55	71473-71240	hypothetical protein	+
56	72351-71470	N-acetylmuramoyl-L-alanine amidase	+
57	73179-72460	soluble lytic murein transglycosylase, lysozyme	
58	74054-73212	PhoH family protein, ribonuclease and ATPase	+
59	74298-74137	hypothetical protein	
60	74709-74308	phage holin	+
61	75171-74728	putative membrane-bound protein	+
62	75613-75164	putative spike protein	+
63	76453-75704	pilus assembly protein	+
64	81691-76493	capsid protein	+
65	82729-81722	L-shaped tail fiber protein	
66	83384-82743	L-shaped tail fiber protein	
67	84613-83600	hypothetical protein	
68	85716-85144	hypothetical protein	
69	86612-85755	bifunctional 5,10-methylene-tetrahydrofolate	
70	87350-86649	dUTPase	+
71	88137-87364	exodeoxyribonuclease III	
72	89374-88238	ATP-dependent DNA ligase	
73	89531-89331	putative ATP synthase	
74	90110-89604	formate/nitrite transporter family protein	
75	90748-90281	hypothetical protein	+
76	91571-91110	NADAR family protein	+
77	92022-91552	antitermination protein, Q-dependent	+
78	92599-91904	hypothetical protein	+
79	93180-92692	LAGLIDADG endonuclease	
80	93618-93457	hypothetical protein	
81	94174-93893	hypothetical protein	+
82	94734-94249	TFIIB zinc-binding	
83	95244-94738	hypothetical protein	
84	97278-95335	hypothetical protein	
85	97638-97294	hypothetical protein	
86	97752-97665	tRNA-Ser-GCT	
87	98810-97884	glutamine dependent NAD+ synthetase	
88	99230-98811	polynucleotide kinase	
89	100822-99224	nicotinamide phosphoribosyltransferase	
90	101784-100819	ribose-phosphate pyrophosphokinase	
91	102326-101781	cysteine hydrolase	
92	102846-102565	probable ATP-dependent amine/thiol ligase family	
93	103249-102863	hypothetical protein	
94	103359-103285	tRNA-Glu-TTC	
95	103670-103597	tRNA-Asp-GTC	
96	103878-103795	tRNA-Leu-GAG	
97	104277-104203	tRNA-Leu-CAG	
98	104450-104380	tRNA-Gln-CTG	
99	104620-104453	hypothetical protein	
100	104915-104676	hypothetical protein	
101	105166-104912	hypothetical protein	
102	105262-105189	tRNA-Arg-ACG	
103	105720-105649	tRNA-His-GTG	

104	105998-105925	tRNA-Lys-CTT	
105	106212-106140	tRNA-Phe-GAA	
106	106321-106250	tRNA-Arg-TCT	
107	106565-106326	phosphocarrier protein HPr	
108	106673-106600	tRNA-Cys-GCA	
109	106878-106804	tRNA-Glu-CTC	
110	107323-107243	tRNA-Thr-TGT	
111	107942-107869	tRNA-Val-TAC	
112	108273-108200	tRNA-Leu-TAG	
113	108664-108368	hypothetical protein	
114	108766-108694	tRNA-Ala-TGC	
115	108899-108825	tRNA-Asn-GTT	
116	109072-108993	tRNA-Pro-GGG	
117	109151-109079	tRNA-Pro-TGG	
118	109481-109209	hypothetical protein	
119	109788-109715	tRNA-Met-CAT	
120	109928-109855	tRNA-Met-CAT	
121	110557-110042	hypothetical protein	
122	111264-110542	hypothetical protein	
123	111368-111286	tRNA-Trp-CCA	
124	111708-111635	tRNA-Ile-GAT	
125	112012-111940	tRNA-Gly-GCC	
126	112478-112077	hypothetical protein	
127	113013-112459	prokaryotic membrane lipoprotein	
128	113118-113045	tRNA-Gly-TCC	
129	113474-113402	tRNA-Lys-TTT	
130	113869-113594	hypothetical protein	
131	114089-113862	hypothetical protein	
132	114529-114164	hypothetical protein	
133	115025-114546	hypothetical protein	+
134	115154-115081	tRNA-Met-CAT	
135	115233-115161	tRNA-Gln-TTG	
136	115939-115391	hypothetical protein	
137	116272-115958	hypothetical protein	
138	117193-116363	nucleotidyltransferase-like protein	
139	117678-117202	hypothetical protein	
140	118074-117733	hypothetical protein	
141	118597-118076	CYTH-like phosphatases	
142	118930-118607	hypothetical protein	
143	119364-118930	hypothetical protein	
144	120110-119526	metallophosphoesterase family protein	
145	120583-120107	hypothetical protein	
146	120964-120641	hypothetical protein	
147	121272-121018	hypothetical protein	
148	121935-121285	P-loop containing nucleoside triphosphate hydrolase	
149	122379-122098	hypothetical protein	
150	122640-122461	hypothetical protein	
151	124046-122748	putative peptidoglycan endopeptidase	
152	125244-124264	hypothetical protein	+
153	125623-125258	hypothetical protein	
154	126393-126097	hypothetical protein	
155	127085-126633	hypothetical protein	
156	128443-127604	hypothetical protein	+

157	129932-128928	exoribonuclease	+
158	130629-129997	hypothetical protein	
159	131186-130914	hypothetical protein	
160	131409-131239	hypothetical protein	
161	131601-131413	hypothetical protein	+
162	132006-131608	hypothetical protein	+
163	132218-132006	hypothetical protein	
164	132769-132305	phosphatase	
165	133059-132799	hypothetical protein	
166	133911-133132	RNAse	
167	134423-134010	hypothetical protein	
168	134903-134472	hypothetical protein	
169	135366-134887	hypothetical protein	
170	136046-135342	hypothetical protein	
171	136356-136180	hypothetical protein	
172	136852-136484	hypothetical protein	
173	137117-136920	hypothetical protein	
174	137645-137271	hypothetical protein	
175	138150-137851	hypothetical protein	
