# Supplementary material for: Genomes of a Novel Group of Phages That Use Alternative Genetic Code Found in Human Gut Viromes
Source: Int J Mol Sci. 2023 Oct 18;24(20):15302. doi: 10.3390/ijms242015302 (PMC10607447; doi:10.3390/ijms242015302)
Supplement: Supplementary file 1 [file ijms-24-15302-s001.zip › Figure S1.pdf]

```
        10        20        30        40        50        60        70        80
....|....|....|....|....|....|....|....|....|....|....|....|....|....|....|....|
MKWAIREAIDVYFKAKSVFQLGAKTFRAGEPVLIFDTVKTSTLEVAAEVSYVTGGRGNARLLSYEGDKTLTFNFEDALLS BK025033_ct6IQ4
................................................................................ BK046881_ctckW2
...................T............................................................ phAss-1_protein1
-------------------------------------------------------------------------------- phAss-1_protein2

        90       100       110       120       130       140       150       160
....|....|....|....|....|....|....|....|....|....|....|....|....|....|....|....|
NEGLAILSGADLIPARNKHLPGAHPDARSVIAHYTEKYSVATNNMRDEDQTKNVYDDDTSLYPAGGPDDPDG------GQ BK025033_ct6IQ4
........................................................................------.. BK046881_ctckW2
......................S.....................QI.T..................AG..VTGDETEH.T phAss-1_protein1
-------------------------------------------------------------------------------- phAss-1_protein2

       170       180       190       200       210       220       230       240
....|....|....|....|....|....|....|....|....|....|....|....|....|....|....|....|
GVGKYAPRGGIDNVWLTRKPYVGQNASIYVMLLDDAGEISGMPVQINLETDDSAEGADKYAYLRKFHTQNDFIAFDLYNK BK025033_ct6IQ4
..........V...........................................................E......... BK046881_ctckW2
VK.T......VN.I.............................L.....ANEDDDEFKRHS..C..KS.DS.V......N phAss-1_protein1
-------------------------------------------------------------------------------- phAss-1_protein2

       250       260       270       280       290       300       310       320
....|....|....|....|....|....|....|....|....|....|....|....|....|....|....|....|
PMSTAEYPDPETIEEAAIFDDQVAYYVDYESC--VRNWVTAWGEKTDYRRVITAPNYGETWGGTMQEQDDGTMSLKGFGH BK025033_ct6IQ4
........N.D.....................--..........................................D..C BK046881_ctckW2
...VE.....IGKDACGV...........DTVSAEG.......DAAQ.M.T.S.A....F..D.KE-----------NSL phAss-1_protein1
-------------------------------------------------------------------------------- phAss-1_protein2

       330       340       350       360       370       380       390       400
....|....|....|....|....|....|....|....|....|....|....|....|....|....|....|....|
YAYLLAPSGGIAQPKAYKEGSDFVYKVNVPSILYQDIVLLDYYVEYTHDATQVSILPDKFGPYMYVEGSSLVRRASDGVD BK025033_ct6IQ4
................................................................................ BK046881_ctckW2
.S..............F............................................A.F................ phAss-1_protein1
-------------------------------------------------------------------------------- phAss-1_protein2

       410       420       430       440       450       460       470       480
....|....|....|....|....|....|....|....|....|....|....|....|....|....|....|....|
LPVEFVIPKFKITTALTFTLAATGDPSTFTFSGDAYPDFSKFDLTRKVLADIQILDADDNYDGASSGIATADPTSYRRFK BK025033_ct6IQ4
................................................................................ BK046881_ctckW2
...........V...................................................G.A....S.......Y. phAss-1_protein1
-------------------------------------------------------------------------------- phAss-1_protein2

       490       500       510       520       530       540       550       560
....|....|....|....|....|....|....|....|....|....|....|....|....|....|....|....|
YNNDSNGEYIWKDRSLEPHQNMDYSDTGNWP--------DKQYNQDAGGPATLTPGSGLIDQENPKIDVDLNDLGTKVSD BK025033_ct6IQ4
...............................--------......................................... BK046881_ctckW2
....TD...L...........L......TMGAGHLSSENY.RF.......R.I...Q....TKP---------------- phAss-1_protein1
-------------------------------------------------------------------------------- phAss-1_protein2

       570       580       590       600       610       620       630       640
....|....|....|....|....|....|....|....|....|....|....|....|....|....|....|....|
VIANAPAGSTLKFNEGIISERLVIDKNLTLEGTTEDGKETILQGGAQLAANGEPVRLTIKNMTLVPDANTPIGVTSQNQ- BK025033_ct6IQ4
...............................................................................- BK046881_ctckW2
-------------------------------------------------------------------------------- phAss-1_protein1
-L.D......IQLSD.SV..K.TV..DI..Q....E..S...EQ.IT..S.N..ISV.V.....Q---.GSF.LHDN.NG phAss-1_protein2

       650       660       670       680       690       700       710       720
....|....|....|....|....|....|....|....|....|....|....|....|....|....|....|....|
-TSTDQRDATVIIENSTIRDFTGKAVYVTDAKTTAIRKSTFENCATGEDTGTVGDHTIDFNLVGVQGANIDLDTVIFKGM BK025033_ct6IQ4
-..V..................S.............C.T........T.......Y.V...................... BK046881_ctckW2
-------------------------------------------------------------------------------- phAss-1_protein1
LEANGT.N.YLTF.DCV.K.....G..TA..RVFKMKNCKI......T...IA..YAV.L..I..KS.VVE.ENCE.V.Y phAss-1_protein2

       730       740       750       760       770       780       790       800
....|....|....|....|....|....|....|....|....|....|....|....|....|....|....|....|
NGHKSSVKVTQRGGPSDKGAGDIPMDIPQATIANFTMSNCDFQTDGQGTPVDLIIGSDHKTPDQPELKNTTGDFPVMISG BK025033_ct6IQ4
....A................................................R..T.....AE................ BK046881_ctckW2
-------------------------------------------------------------------------------. phAss-1_protein1
C.A.AAF..........E........KG.SY.N.V.ITG.S.D..---....VRL.TE.......D.E...AN....... phAss-1_protein2

       810       820       830       840       850       860       870       880
....|....|....|....|....|....|....|....|....|....|....|....|....|....|....|....|
NKTPVRVQNLYKGNDDVVTVPAGATGYKNAKSDFVVVGAATP---------VTPISINAAGYDDIYCAFRAAKAGDTVTL BK025033_ct6IQ4
.........P....E...........................---------.........................V.V. BK046881_ctckW2
D.N.---------------------------------------------------------------------------- phAss-1_protein1
...EIN.HVAPT.KSY.--..V.G.....GTGE.T.I.GT..EPDPDPEEPT.GA..GDVD.AT.PE.VA.L.D...LVF phAss-1_protein2
```

```
       890       900       910       920       930       940       950       960
....|....|....|....|....|....|....|....|....|....|....|....|....|....|....|....|
NQDIAISDNDVNIAGDSMVAAMIPAGVTLDGNGHKITVGTITG-------KHILGVNGENVTIK-NVTIEGAAGAKSGVV BK025033_ct6IQ4
.E......D..................................-------..............-......TT....... BK046881_ctckW2
-------------------------------------------------------------------------------- phAss-1_protein1
...YD---QPII.D.---------VD..INL...T.ANTAPVYDPAGGIISL.AIE..A....TG.G..HAL.DDCYC.D phAss-1_protein2

       970       980       990      1000      1010      1020      1030      1040
....|....|....|....|....|....|....|....|....|....|....|....|....|....|....|....|
VSGVSAKLTATNVTINNCANCGIQ----VTNGGQVILDNYKSNGN---AWGSVNCDKG---AGGATPRV---TFNSGAMA BK025033_ct6IQ4
........................----.................---T.........---T.......---........ BK046881_ctckW2
-------------------------------------------------------------------------------- phAss-1_protein1
.QNG.MLTIEDGTF.G.ITA.YV.QGDL.IE..DYSIQQLSEP..GGDERFTL..LDSAYKD.T..IE.SGG...KFDP. phAss-1_protein2

      1050      1060      1070      1080      1090      1100      1110
....|....|....|....|....|....|....|....|....|....|....|....|....|....|....|....
ENVEIYTELVDIQCVTAPSLTEVIGVGDTLKGFKYYTSDMARLGVAAVVVDGKTTVYEDMVEAEEAADEAGVDVVI--L BK025033_ct6IQ4
.............................................................K.K............--. BK046881_ctckW2
------------------------------------------------------------------------------- phAss-1_protein1
N.L-----------------A.--.P.TNFCA------------------------------.GYTTEQK.DKYTVIKA phAss-1_protein2
```

Figure S1: Amino acid sequence alignment of the phAss-1, BK046881_ctckW2, and BK025033_ct6IQ4 tail fiber proteins.
