# Supplementary material for: Genomes of a Novel Group of Phages That Use Alternative Genetic Code Found in Human Gut Viromes
Source: Int J Mol Sci. 2023 Oct 18;24(20):15302. doi: 10.3390/ijms242015302 (PMC10607447; doi:10.3390/ijms242015302)
Supplement: Supplementary file 1 [file ijms-24-15302-s001.zip › Figure S2.pdf]

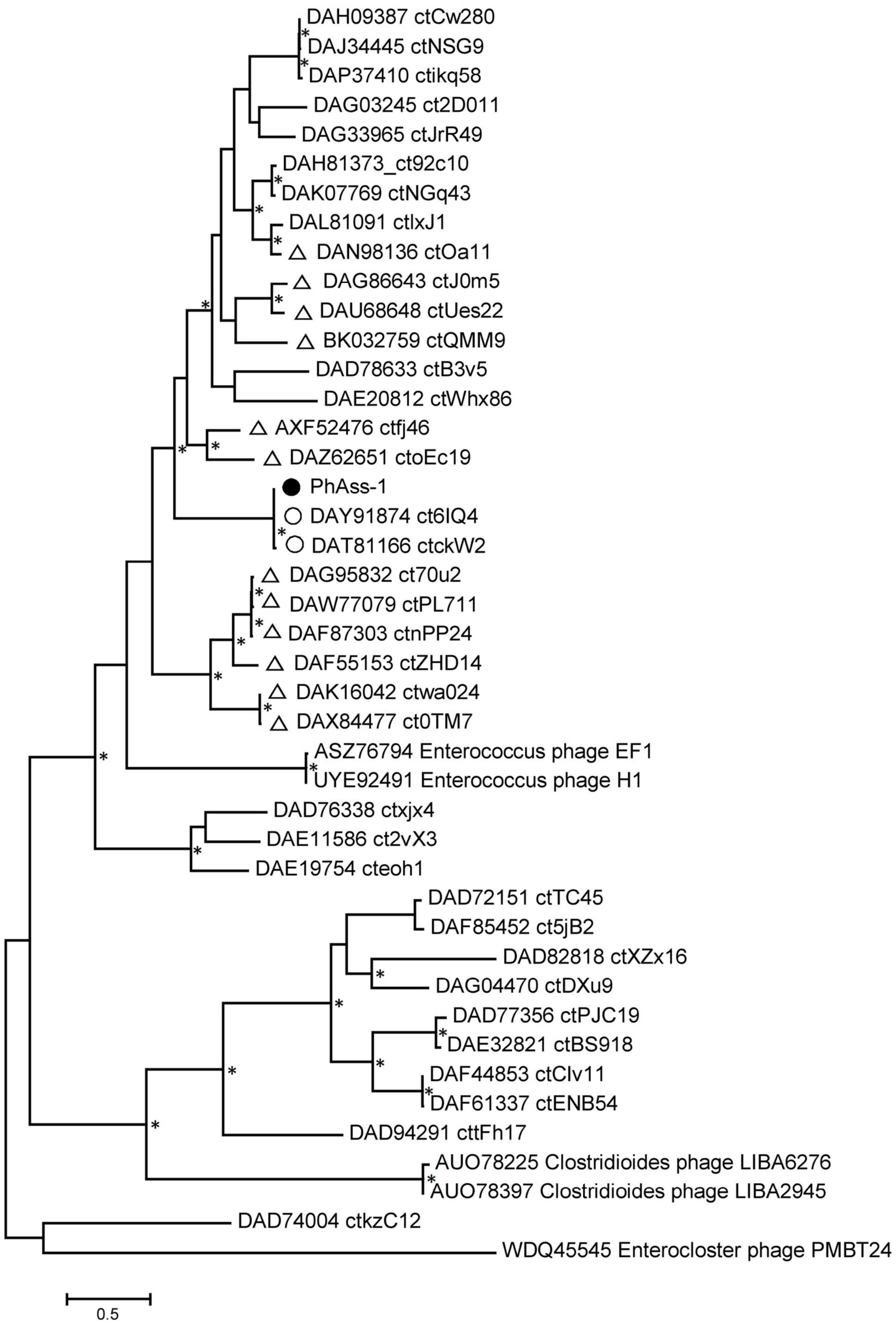

Figure S2: Maximum Likelihood phylogenetic tree of the phAss-1 major capsid protein generated using IQ-tree software. The investigated sequence of phAss-1 is marked with a black circle; corresponding sequences of relative phages from the proposed *Phassvirus* genus are indicated with empty circles; sequences from phage genomes that are listed in Table are marked with empty triangles. Nodes with 95% statistical significance are marked with asterisks calculated from 1000 ultrafast bootstrap (UFBOOT) replicates. The scale bar represents the number of substitutions per site.
