# Supplementary material for: Genomes of a Novel Group of Phages That Use Alternative Genetic Code Found in Human Gut Viromes
Source: Int J Mol Sci. 2023 Oct 18;24(20):15302. doi: 10.3390/ijms242015302 (PMC10607447; doi:10.3390/ijms242015302)
Supplement: Supplementary file 1 [file ijms-24-15302-s001.zip › Figure S5.pdf]

```
        10        20        30        40        50        60        70        80
....|....|....|....|....|....|....|....|....|....|....|....|....|....|....|....|
MGYQLVKHLESQQYNRKLFYLDQGAEDLPPITNFTDIQPGSEARSLTTGEKWILNTHFKWVYIKESGCGCCGGSSGAGGG phAss-1
................................................................................ BK046881_ctckW2
..F............................................................................. BK025033_ct6IQ4

        90       100       110       120       130       140       150       160
....|....|....|....|....|....|....|....|....|....|....|....|....|....|....|....|
DAGGDGTGNTPGGGTEVVDPDPVPGEEPTIEGITLSPLNITVGQGATVAFTAVVQGNAQLSRGVKWTIKGQRVSDTTITQ phAss-1
G...----......--T............................................................... BK046881_ctckW2
N.....S......................................................................... BK025033_ct6IQ4

       170       180       190       200       210       220       230       240
....|....|....|....|....|....|....|....|....|....|....|....|....|....|....|....|
DGILKIGEKETSKTITVRVTSEADESVYAQAVVSVDVNMEDPLAPMVTGIVLVPTDVEVVLGRSVMFNTMVNGVNLSDFS phAss-1
.............................................V.................................. BK046881_ctckW2
.............................................V.................................. BK025033_ct6IQ4

       250       260       270       280       290       300       310       320
....|....|....|....|....|....|....|....|....|....|....|....|....|....|....|....|
AVYSVSGQSSTNTYINQDGTLHVGADEQSKVLVVTAKAAADQRFFATATVSVTDSQHAVDQSTVTEVIVYPGACQIGCGY phAss-1
.............H.D....................................................I....T...... BK046881_ctckW2
.........................................................................T...... BK025033_ct6IQ4

       330       340       350       360       370       380       390       400
....|....|....|....|....|....|....|....|....|....|....|....|....|....|....|....|
SQQFAAKVNGVNNPSQQVVWKLTGATSKDTCVTPNGLVFVGEDEKSNMLVLTAYSQKTPEVYGEAIIDVVPAETPGVDEV phAss-1
..............................R................................................. BK046881_ctckW2
...........................S..R..S.............................................. BK025033_ct6IQ4

       410       420       430       440       450       460       470       480
....|....|....|....|....|....|....|....|....|....|....|....|....|....|....|....|
TVDAIIITPDLVELEQGWQTVFKAVVIGKNNPSQAVTWSLTGNNVQTTYLTDDGVLTIGIGETAKSLQVRATSKQDTTKY phAss-1
..........M.........................................E........................... BK046881_ctckW2
..........M.............................................S.......Q............... BK025033_ct6IQ4

       490       500       510       520       530       540       550       560
....|....|....|....|....|....|....|....|....|....|....|....|....|....|....|....|
NIAYVTIAAYDAGGDNGFTDVPATPLNTKYVRERTANGSAVWTPIEEEVEEPIPEPDPEINSVEVSPNAVTVAPGSVITF phAss-1
......V..........................................K.............................. BK046881_ctckW2
....................I.....................................V..................... BK025033_ct6IQ4

       570       580       590       600       610       620       630       640
....|....|....|....|....|....|....|....|....|....|....|....|....|....|....|....|
AAIVNGSEELSKEVTWSISGQRDPNTKITSDGVLTIGADEDAMMIRVTARSIVDTSKYGTATISIDEEAPVLQQVTGFYL phAss-1
....................................................I.................I......... BK046881_ctckW2
................................................................................ BK025033_ct6IQ4

       650       660       670       680       690       700       710       720
....|....|....|....|....|....|....|....|....|....|....|....|....|....|....|....|
EPIEATVIKDHSLRFQAIVTGVNITNHDATFTVSGNQSPQTIITPEGVLYVDKEETSALLIVTGTCAVDPKFTDTSLVTV phAss-1
...............................A...................................A..N.......A. BK046881_ctckW2
...........................E...A......R........................A...A..N......... BK025033_ct6IQ4

       730       740       750       760       770       780       790       800
....|....|....|....|....|....|....|....|....|....|....|....|....|....|....|....|
IPPELAEDEPVVTVIQLYPAYTQIGRGMSARFAVQLTGLNNPPASIIWDLTGATSLATHVSRDGVVYIGADEQLHEITLR phAss-1
............................N................................................... BK046881_ctckW2
.....................A...............................................V.......... BK025033_ct6IQ4

       810       820       830       840       850       860       870       880
....|....|....|....|....|....|....|....|....|....|....|....|....|....|....|....|
ATVSYDPTKFAEATINVVSEDTPGIDETTVDAVIISPTAVESDPGHRITFKATVIGQNNPSQEVIWSLDGNLKAETTINQ phAss-1
.....................................A.......................................... BK046881_ctckW2
............S.............K..........A................................S......... BK025033_ct6IQ4

       890       900       910       920       930       940       950       960
....|....|....|....|....|....|....|....|....|....|....|....|....|....|....|....|
MGVLTIATDETARVLKITATSVADSTVKSTSYVTISKTQDTPETGIEDVPNDPLNMNYQRRIDENGRTYWVKYPEVGNDG phAss-1
................................................................................ BK046881_ctckW2
..........................................D...........................I......... BK025033_ct6IQ4

       970       980       990      1000      1010      1020      1030      1040
....|....|....|....|....|....|....|....|....|....|....|....|....|....|....|....|
QRYMRRYNQVTGEYEWEPYPEIPLDGKQYARQYNPETRTVEWVEVEASGGGRPDAPINLGTVGTKAELDRFEIPADSMDG phAss-1
...................................L............................................ BK046881_ctckW2
..................................AL..K......................................... BK025033_ct6IQ4
```

```
      1050      1060      1070      1080      1090      1100      1110      1120
....|....|....|....|....|....|....|....|....|....|....|....|....|....|....|....|
DFIFVEEDESQ------------------------------------------------------------------GHC phAss-1
...Y.......DYCSTMYIVQSGPDGRKEFVLSMVFGRPGEPINLGTVGTKAELDEFDIPANSIDGDFIYVENDETQ... BK046881_ctckW2
...........NHCPAMYIVQTGPDGQKKFVLSMVFGRPAAPINLGTLGTKAELDEFDIPANSMDGDFIYVENDETQ... BK025033_ct6IQ4

      1130      1140      1150      1160      1170      1180      1190      1200
....|....|....|....|....|....|....|....|....|....|....|....|....|....|....|....|
PTMYIVQTNERGEKEFVLSMVFGRKPILGDKLDILYVLDNSVHSQLIKDIEVLSEFDTNEKRYELYKSPSAWHLVTELHE phAss-1
......H.................................GI...................................... BK046881_ctckW2
......H......................................................................... BK025033_ct6IQ4

      1210      1220      1230      1240      1250      1260      1270      1280
....|....|....|....|....|....|....|....|....|....|....|....|....|....|....|....|
GFWKEVMKHPSWYKMLIGMARDDGSTKAYITCFTDEHEFEHYKTQDIDNPDPIQNTLAWTNTVQPGNVFTAANSSRLVQY phAss-1
................V....A....R...............................................T..I.. BK046881_ctckW2
..........D.....V........................................................AT..I.. BK025033_ct6IQ4

      1290      1300      1310      1320      1330      1340      1350      1360
....|....|....|....|....|....|....|....|....|....|....|....|....|....|....|....|
LFHLDTMSLTVGSQEEIPPGTEPGVTPWATVIGEYSTLINGETIGPLADVTDPTGYVWVSEHEKFMIGSAGDGSQMFCYN phAss-1
................................................................................ BK046881_ctckW2
..................................................................L....N........ BK025033_ct6IQ4

      1370      1380      1390      1400      1410      1420      1430      1440
....|....|....|....|....|....|....|....|....|....|....|....|....|....|....|....|
IDTGDHFIFNVPNYSRESIWSTEVPYSLATDVNERYFFMYITTTLGIWGDRVTHETKEIHWNANPYSGLSDPGQLTKPGI phAss-1
.........D......K..PN....F...................................................... BK046881_ctckW2
.........D......DH.PN....F....................V..........Q.T.RPTG.........Y..... BK025033_ct6IQ4

      1450      1460      1470      1480      1490      1500      1510      1520
....|....|....|....|....|....|....|....|....|....|....|....|....|....|....|....|
SPNGNYYMHTSTNPETPTFTSFSFVTGRLVTEAPIDGTSSSAICLNNNVVFTNTPEGRIITYNFDPVRGTLTVRAHGDDN phAss-1
........................K..........N.........................................S.. BK046881_ctckW2
D.T.KF...........A......D..NI...G............................................N.. BK025033_ct6IQ4

      1530      1540      1550      1560      1570      1580      1590      1600
....|....|....|....|....|....|....|....|....|....|....|....|....|....|....|....|
YTPHACRYVVEATTNGNQVLCVKVERGVNCPVWFVYDCDRDTIIAESTNREENEYCSQTNPQAKSCTYELPTSAGERYLL phAss-1
...Y.....................A.E......................GR.D.....S.............V...... BK046881_ctckW2
.........................A.A...........S.......A................................ BK025033_ct6IQ4

      1610      1620      1630      1640      1650      1660      1670      1680
....|....|....|....|....|....|....|....|....|....|....|....|....|....|....|....|
SSATTQQGLVLSYDGSTWKEVTIPFGGLDNTKHEYNQPILMNDGDILVTQDDTGKPVGFDLVKMEVVDLDEVLHPGGDAH phAss-1
...............N...............................I...EH.N..............E.......... BK046881_ctckW2
.....K.......E.G...............................I.....................E.......... BK025033_ct6IQ4

      1690      1700      1710      1720      1730
....|....|....|....|....|....|....|....|....|....|..
MVQLDDTHFMWCTDEGSQLLRSKGDGTQEVVLELPEQQWIVFGFGRRSESGG     phAss-1
..............D........E.......................TG...     BK046881_ctckW2
..............D........E......I..M.............TG...     BK025033_ct6IQ4
```

Figure S5: Amino acid sequence alignment of the phAss-1, BK046881_ctckW2, and BK025033_ct6IQ4 capsid proteins.
